# Regional Right Ventricular Function Assessed by Intraoperative Three-Dimensional Echocardiography Is Associated With Short-Term Outcomes of Patients Undergoing Cardiac Surgery

**DOI:** 10.3389/fcvm.2022.821831

**Published:** 2022-03-22

**Authors:** Marius Keller, Marcia-Marleen Duerr, Tim Heller, Andreas Koerner, Christian Schlensak, Peter Rosenberger, Harry Magunia

**Affiliations:** ^1^Department of Anesthesiology and Intensive Care Medicine, University Hospital Tuebingen, Eberhard-Karls-University, Tuebingen, Germany; ^2^Department of Thoracic and Cardiovascular Surgery, University Hospital Tuebingen, Eberhard-Karls-University, Tuebingen, Germany

**Keywords:** right ventricle, ejection fraction, cardiac surgery, outcome research, three-dimensional echocardiography

## Abstract

**Background:**

The assessment of right ventricular (RV) function in patients undergoing elective cardiac surgery is paramount for providing optimal perioperative care. The role of regional RV function assessment employing sophisticated state-of-the-art cardiac imaging modalities has not been investigated in this cohort. Hence, this study investigated the association of 3D echocardiography-based regional RV volumetry with short-term outcomes.

**Materials and Methods:**

In a retrospective single-center study, patients undergoing elective cardiac surgery were included if they underwent 3D transesophageal echocardiography prior to thoracotomy. A dedicated software quantified regional RV volumes of the inflow tract, apical body and RV outflow tract employing meshes derived from 3D speckle-tracking. Echocardiographic, clinical and laboratory data were entered into univariable and multivariable logistic regression analyses to determine association with the endpoint (in-hospital mortality or the need for extracorporeal circulatory support).

**Results:**

Out of 357 included patients, 25 (7%) reached the endpoint. Inflow RV ejection fraction (RVEF, 32 ± 8% vs. 37 ± 11%, *p* = 0.01) and relative stroke volume (rel. SV) were significantly lower in patients who reached the endpoint (44 ± 8 vs. 48 ± 9%, *p* = 0.02), while the rel. SV of the apex was higher (38 ± 10% vs. 33 ± 8%, *p* = 0.01). Global left and right ventricular function including RVEF and left ventricular global longitudinal strain did not differ. In univariable logistic regression, tricuspid regurgitation grade ≥ 2 [odds ratio (OR) 4.24 (1.66–10.84), *p* < 0.01], inflow RVEF [OR 0.95 (0.92–0.99), *p* = 0.01], inflow rel. SV [OR 0.94 (0.90–0.99), *p* = 0.02], apex rel. SV [OR 1.07 (1.02–1.13), *p* < 0.01] and apex to inflow rel. SV ratio [OR 5.81 (1.90–17.77), *p* < 0.01] were significantly associated with the endpoint. In a multivariable model, only the presence of tricuspid regurgitation [OR 4.24 (1.66–10.84), *p* < 0.01] and apex to inflow rel. SV ratio [OR 6.55 (2.09–20.60), *p* < 0.001] were independently associated with the endpoint.

**Conclusions:**

Regional RV function is associated with short-term outcomes in patients undergoing elective cardiac surgery and might be helpful for optimizing risk stratification.

## Introduction

Perioperative right ventricular (RV) dysfunction dramatically limits patient outcomes following cardiac surgery (1, 2). While most current risk stratification scores disregard baseline RV function, even subclinical preoperative RV dysfunction has been shown to be associated with increased postoperative mortality (3). However, as the RV is of a complex anatomical and physiological nature, detecting subtle deviations from normal RV function is challenging. Hence, the hunt for novel non-invasive parameters that could yield further insight into RV pathophysiology is thriving. Echocardiography can be considered the most important non-invasive diagnostic method for quantifying RV function. In perioperative medicine, it is mainly applied by cardiologists via transthoracic acoustic windows distinctively before referral to surgery and by cardiac anesthesiologists in the operating room using transesophageal echocardiography (TEE). Intraoperative TEE has the potential to influence surgical decision-making, and its application is associated with improved outcomes (4, 5). Due to the ability to generate high-resolution images under general anesthesia, TEE facilitates the acquisition of 3D datasets that are advantageous over 2D recordings in the assessment of baseline RV function (6, 7). 3D volumetric analyses of the RV, especially 3D-derived RV ejection fraction (RVEF), carry incremental prognostic information in various chronic cardiac diseases (8). Depicting global into regional systolic RV function is a promising approach for unveiling contraction patterns associated with early pathological remodeling or abnormal loading conditions and hence increasing sensitivity in detecting RV dysfunction (7, 9–11). Literature on regional RV systolic function using 3D echocardiography regarding short-term prognosis after cardiac surgery is sparse (12). Therefore, the present study investigated the association of regional systolic function of the right ventricle with short-term outcome in patients undergoing cardiac surgery using mesh models derived from intraoperative transesophageal 3D-STE and a previously reported software approach (13). The performances of the novel parameters were compared to established clinical and echocardiographic measures that are routinely utilized for risk stratification. To our knowledge, this is the first study to investigate sophisticated 3D-derived intraoperative regional RV volumetry in patients undergoing cardiac surgery.

## Materials and Methods

### Patients

Ethical approval for retrospective patient inclusion was granted by the Ethics Committee of the Medical School at the University of Tuebingen (Trial Registration # IRB 350/2015R) and the study was conducted in accordance with the Declaration of Helsinki. Patients were screened for inclusion to this single-center cohort study if they underwent one of the following elective cardiac surgery procedures between November 2013 and October 2018: on-pump coronary artery bypass grafting, off-pump coronary artery bypass grafting (OPCAB), left-sided valve surgery, thoracic aortic surgery or left ventricular assist device (LVAD) implantation. Surgeries were categorized as “mixed” if one of the major procedures (except LVAD implantation) was combined with other procedures, or a combination of two or more major procedures (except LVAD implantation) was performed. Of the screened patients, only those with standardized intraoperative 3D transesophageal echocardiograms available in the institutional database were included in the study. Patients were excluded if they were underage (<18 years) at the time of surgery, needed extracorporeal circulatory support preoperatively or electronic patient records were incomplete for clinical data analysis.

### Anesthesia and Intraoperative Echocardiography

The institutional regimen for standardized anesthesia during cardiac surgery and intraoperative echocardiography was described previously (14, 15). Briefly, anesthesia was induced with midazolam, sufentanil and rocuronium. After endotracheal intubation, anesthesia was maintained with sevoflurane and continuous administration of sufentanil. A norepinephrine infusion was titrated, if necessary, to maintain a mean arterial pressure above 65 mmHg. No additional sympathomimetics were used between anesthesia induction and thoracotomy. Inotropic therapies initiated prior to anesthesia induction were maintained unaltered.

The institutional standard for intraoperative TEE requires recordings after the establishment of hemodynamic stability following anesthesia induction but prior to thoracotomy. TEE was performed by specially trained cardiac anesthesiologists using commercially available 3D-compatible probes (Philips X7-2t Matrix, Philips Healthcare, Inc., Andover, MA, USA and Siemens Z6Ms TEE probe, Siemens Healthineers AG, Erlangen, Germany). Representative 3D loops of the fully projected right and left ventricles at a frame rate above 20 fps were acquired.

### Established Echocardiographic Parameters of LV and RV Function

All functional parameters were derived offline from the intraoperatively acquired 3D TEE studies using commercially available 3D-STE software packages. Left ventricular volumes, ejection fraction (LVEF) and global longitudinal strain (LV-GLS) were calculated using 4D LV-ANALYSIS software (Tomtec Imaging Systems GmbH, Unterschleissheim, Germany). If 3D LV datasets of sufficient quality were not available, left ventricular volumes, LVEF and LV-GLS were derived from two-dimensional recordings using appropriate speckle-tracking software. RV volumes, RVEF, fractional area change (FAC) and tricuspid annular plane systolic excursion (TAPSE) were calculated with the widely used 4D RV-Function software (version 2.0, Tomtec Imaging Systems GmbH, Unterschleissheim, Germany). Image acquisition and measurements were carried out in accordance with guideline recommendations (16–18).

### Regional Right Ventricular Volumetry

After exporting the endocardial RV mesh files derived from 3D-STE (UCD file format), offline regional volumetry was performed using custom-made post-processing software as previously reported (13). The software algorithm is written in C++ based on the Visualization Toolkit (Ver. 7.1.1, Kitware, Inc., Clifton Park, New York, USA) and automatically performs regional volumetry of three RV regions:

- “inflow”—comprising mainly the anatomic region of the tricuspid annulus leaflets, also referred to as the “inlet.”
- “apex”—comprising the apical trabecular region.
- “RVOT”—comprising the right ventricular outflow tract, also referred to as the “outlet.”

Virtual cutting surfaces derived from an annular set of mesh points around the border of two regions served as the borders to divide the RV into the three regions. A set of two neighboring mesh points and the common center point each formed a triangle, all of which formed the cutting surface. The cutting points were manually defined and evaluated on a set of exemplary meshes, and the same set of cutting points was applied to all meshes analyzed for the study (13). Regional volumetric analysis yielded the *RVEF* (difference between maximum and minimum volume divided by the maximum volume of a region within the cardiac cycle, given in %), *end-diastolic volume (EDV*, maximum volume of a region within the cardiac cycle, given in ml), *end-diastolic volume index* (*EDVi*, normalized to body surface), *stroke volume* (*SV*, difference between maximum and minimum volume of a region within the cardiac cycle) and the *relative stroke volume* (*rel. SV*, relative contribution to the global RV stroke volume, given in %) individually for each region.

### Clinical Data and Outcome

Epidemiologic, laboratory, clinical and outcome data were extracted from electronic patient records. The following baseline parameters were recorded: age, body mass index, body surface, estimated glomerular filtration rate (eGFR), hematocrit, lactate, the presence of chronic lung disease (defined as long-term use of bronchodilators or steroids for lung disease), the presence of pulmonary hypertension (defined as systolic pulmonary artery pressure > 30 mmHg assessed by Doppler echocardiography), the presence of tricuspid regurgitation (defined as grade 2 or 3 measured by color Doppler echocardiographic jet size and RA dimensions), the presence of diabetes (defined as insulin-dependent), the New York Heart Association (NYHA) functional class and the European System for Cardiac Operative Risk Evaluation II (EuroSCORE II). For short-term outcome analysis, the combined endpoint reflecting short-term outcome was defined as a composite of in-hospital mortality and/or the need for extracorporeal life support (ECLS, by venoarterial extracorporeal membrane oxygenation) within the postoperative hospital period.

### Statistical Analysis

Normally distributed continuous variables are presented as the mean ± standard deviation, and non-normally distributed continuous variables are presented as the median (interquartile range). Categorial variables are displayed as absolute numbers and percentages. Normally distributed samples were compared with unpaired Student's *t*-tests, while non-normally distributed samples were compared using Mann–Whitney U tests. Proportions were compared using chi-square tests. Binomial logistic regression was used to examine risk factors that could influence the endpoint, which are depicted with odds ratios (ORs) and their 95% confidence intervals (CIs). Variables that were significantly associated with the endpoint in univariable logistic regression were considered for inclusion in multivariable analysis. Multivariable logistic regression was performed with a forward stepwise conditional selection method (entry inclusion if *p* < 0.05, exclusion if *p* > 0.1) with respect to collinearity and the number of events per variable (19). The omnibus test of model coefficients was used to compare chi-square values of logistic regression models. Reproducibility of global and regional RVEF analyses was evaluated on 25 randomly selected patients. The same investigator reanalyzed those patients at least 3 months after the initial measurements for intraobserver reproducibility analysis, while a second investigator performed measurements for interobserver reproducibility analysis; both were blinded to the initial results. Intraclass correlation coefficients (ICCs) from a two-way random model for absolute agreement on average, Pearson's correlation coefficient r, Bland–Altman bias, and 95% limits of agreement were calculated to quantify reproducibility. *P*-values of <0.05 were regarded as significant. Prism (GraphPad Software, San Diego, CA), MedCalc (MedCalc Software Ltd., Ostend, Belgium) and SPSS (IBM Corp., Armonk, NY) were used for statistical calculations and data presentation.

## Results

### Clinical Data

Out of 3,561 patients in the institutional database screened for inclusion, *n* = 357 patients could be included. Baseline clinical data are displayed in Table 1. On average, patients were 66 ± 13 years old, moderately overweight (mean body mass index 27 ± 5 kg/m^2^) and predominantly male (73%). Chronic lung disease, pulmonary hypertension, tricuspid regurgitation or diabetes were only present in a small number of patients (8, 16, 13, and 10%, respectively). Mixed procedures were most common (28%, details listed in Supplementary Table 1), followed by isolated left-sided valve surgery (27%) and off-pump (19%) and on-pump (14%) coronary artery revascularizations. Isolated surgery of the thoracic aorta (5%) or LVAD implantations (7%) were less common. Thoracic aortic surgery was performed for aneurysm repair (*n* = 16) or resection of an intramural hematoma (*n* = 1) by performing supracoronary ascending aortic replacement (*n* = 8), hybrid frozen elephant trunk procedures (*n* = 4), aortic root and ascending aorta replacement (*n* = 4) or resection of ascending aorta intramural hematoma (*n* = 1). Overall, baseline lactate levels, hematocrit, and eGFR were within the normal ranges.

**Table 1:** Baseline clinical data of the complete patient cohort (*n* = 357).

**Parameter**	**Result**
Age, years	66 ± 13
Male, *n*	259 (73)
Body mass index, kg/m^2^	27 ± 5
Chronic lung disease^*^, *n*	29 (8)
Pulmonary hypertension^†^, *n*	57 (16)
Tricuspid regurgitation^‡^, *n*	45 (13)
Diabetes^§^, *n*	36 (10)
**NYHA functional class**
I	67 (19)
II	105 (29)
III	118 (33)
IV	67 (19)
eGFR, ml/min	80 (61–102)
Lactate, mmol/l	0.90 ± 0.48
Hematocrit, %	37 ± 6
EuroSCORE II, %	3.4 (1.5–7.8)
**Type of surgery**
On-pump coronary artery bypass grafting, *n*	51 (14)
OPCAB, *n*	69 (19)
Left-sided valve surgery, *n*	96 (27)
Thoracic aortic surgery, *n*	17 (5)
LVAD implantation, *n*	25 (7)
Mixed procedures, *n*	99 (28)

*Values are means ± standard deviations, medians (interquartile ranges) or n (%)*.

*ECLS, extracorporeal life support; eGFR, estimated glomerular filtration rate; LVAD, left ventricular assist device; NYHA, New York Heart Association; OPCAB, off-pump coronary artery bypass surgery*.

* *Long term use of bronchodilators or steroids for lung disease*.

† *Systolic pulmonary artery pressure > 30 mmHg*.

‡ *Grade ≥ 2*.

§ *Insulin-dependent*.

### Echocardiographic Data

**Figure 1:**
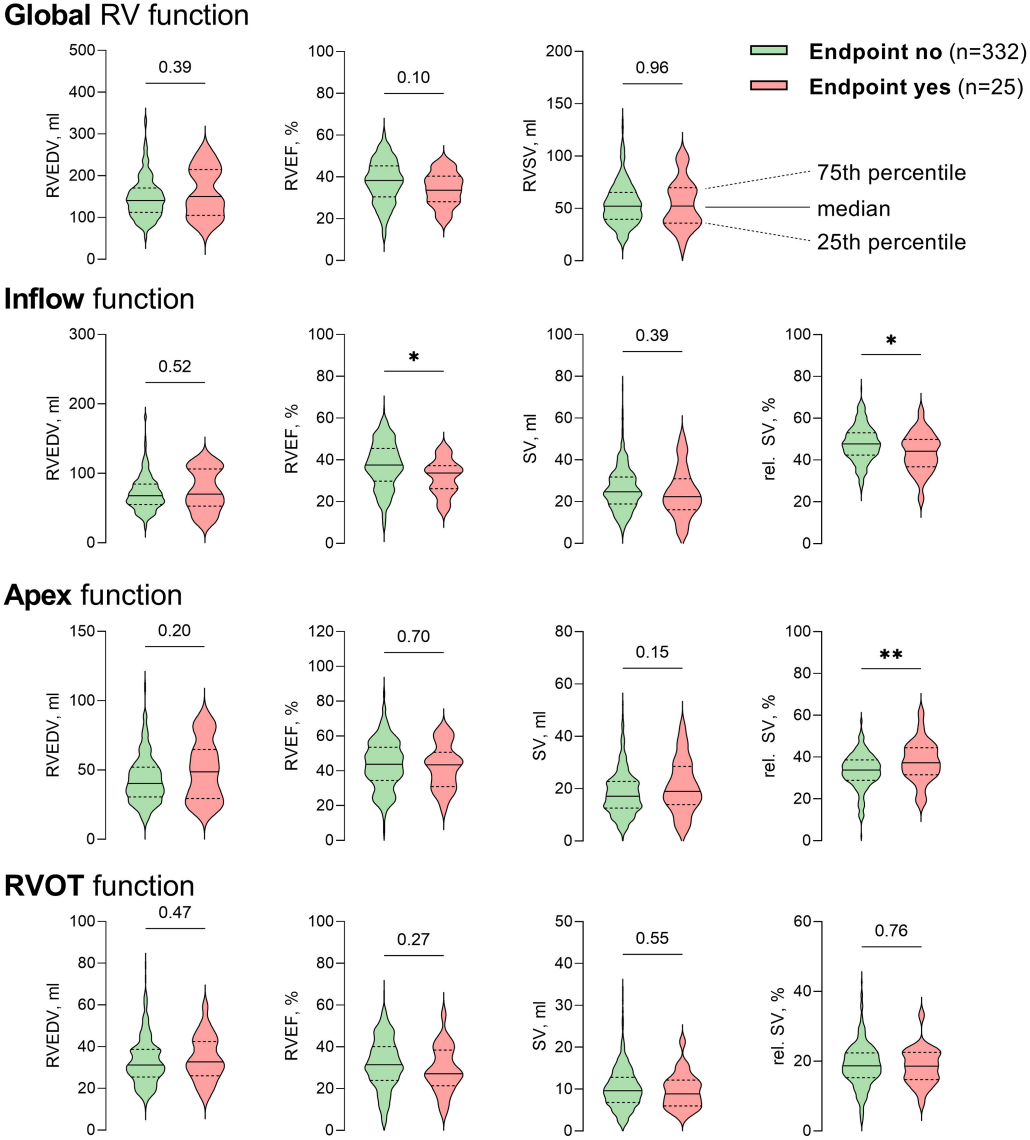
Violin plots of global and regional RV volumetry parameters using 3D-STE-derived mesh models. Global RV function of patients who reached the endpoint (red) did not differ significantly from patients with an uncomplicated postoperative course (green). However, patients who died postoperatively or required extracorporeal circulatory support had lower inflow ejection fraction and inflow relative stroke volume, while relative stroke volume of the apex was significantly higher. *P*-values are derived from unpaired Student's *t*-tests. Rel., relative; RVEDV, right ventricular end-diastolic volume; RVEF, right ventricular ejection fraction; STE, speckle-tracking echocardiography; (RV)SV, (right ventricular) stroke volume; *p < 0.05; **p < 0.01.

**Figure 2:**
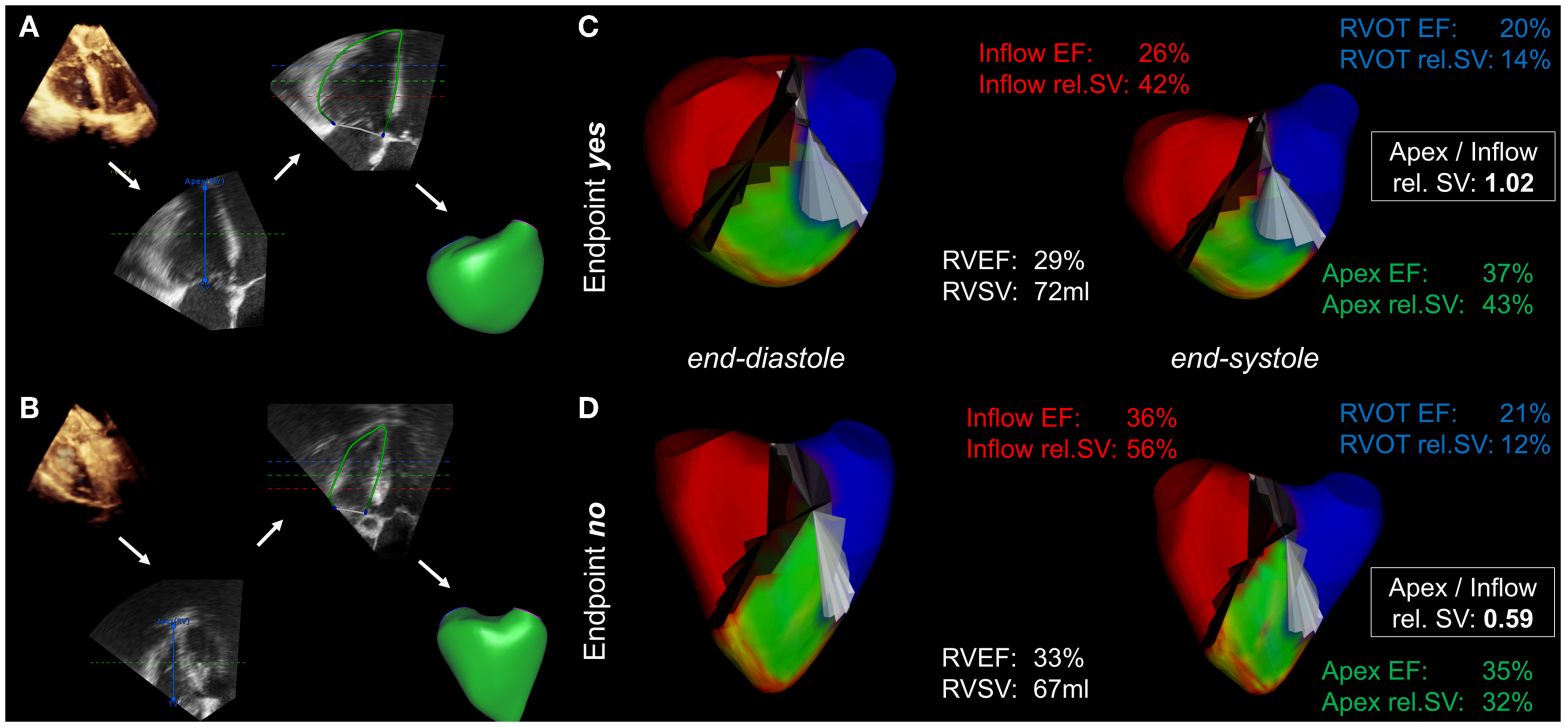
Mesh-derived regional RV volumetry. Visualization of regional RV volumetry in a patient who reached the endpoint **(A,C)** compared with that in a patient with an uneventful postoperative course **(B,D)**. **(A,B)** The intraoperative 3D datasets undergo segmentation and endocardial borders are tracked using speckle-tracking technology, resulting in the generation of mesh models (workflow indicated by the white arrows). **(C,D)** Mesh-derived regional volumetry of the inflow (red), apex (green) and RVOT (blue). The displayed results include regional ejection fractions (EF), relative stroke volumes (rel. SV) and the apex to inflow rel. SV ratio. The cutting planes between the regions are colored black and white.

Echocardiographic parameters of LV and RV systolic function, including mesh-derived regional RV volumetric data (Figure 1) of patients who reached the endpoint compared to patients who did not reach the endpoint, are listed in Table 2. Global left and right ventricular volumes did not differ significantly between the outcome groups. The same relationship was observed for global RV and LV functional parameters derived from 3D-STE (LVEF, LV-GLS, RVEF, RV SV, TAPSE, and FAC). Of note, the percentage of patients with severely decreased LVEF or RVEF (<20% each) was similar in both groups. As a simple measure of longitudinal function of the basal inflow region, 3D-derived TAPSE correlated moderately with inflow RVEF (*r* = 0.50, 95% CI 0.42–0.58, *p* < 0.001). Global and regional RVEF decreased with increased systolic pulmonary artery pressures (Supplementary Table 2). Regional RV volumetry was feasible in all included patients. Patients who reached the endpoint had significantly lower systolic function in the inflow region (reflected by inflow RVEF and rel. SV) and higher rel. SV in the apex region than patients who did not reach the endpoint (Figure 2). Interestingly, the end-diastolic volumes of the three RV regions did not differ significantly. The opposite changes in the rel. SV of the inflow and the rel. SV of the apex comprised a statistically significant difference in the *apex to inflow rel. SV ratio* between the different outcome groups. This parameter was not defined a priori but based on the analysis' findings.

**Table 2:** Conventional and novel echocardiographic parameters derived from transesophageal echocardiography of patients who reached the endpoint (in-hospital mortality or the need for ECLS) and patients who did not reach the endpoint.

**Parameter**	**Endpoint yes (*n* = 25)**	**Endpoint no (*n* = 332)**	***p*-value**
**LV and RV systolic function parameters (derived from 3D-STE)**			
LVEDV, ml	176 (134–213)	150 (117–203)	0.30
LVEDVi, ml/m^2^	91 (74–121)	78 (62–106)	0.23
LVESV, ml	106 (58–143)	90 (65–137)	0.54
LVESVi, ml/m^2^	54 (31–75)	46 (34–70)	0.41
LVEF, %	38 ± 15	38 ± 14	0.47
LVEF <20%, *n*	4 (16)	36 (11)	0.66
LV-GLS, %	−11.6 ± 6.5	−12.6 ± 5.6	0.38
RVEDV, ml	150 (108–213)	141 (112–171)	0.39
RVEDVi, ml/m^2^	78 (55–99)	73 (61–87)	0.25
RVESV, ml	94 (73–139)	87 (65–110)	0.15
RVESVi, ml/m^2^	51 (38–67)	44 (34–57)	0.08
RVEF, %	34 ± 8	38 ± 10	0.10
RVEF <20%, *n*	1 (4)	16 (5)	0.76
RV SV global, ml	54 ± 23	54 ± 20	0.96
TAPSE, mm	11 ± 6	12 ± 6	0.58
FAC, %	27 ± 8	31 ± 10	0.08
**Mesh-derived regional RV volumetry**			
RVEF inflow, %	32 ± 8	37 ± 11	**0.01**
EDV inflow, ml	70 (54–106)	68 (55–85)	0.52
EDVi inflow, ml/m^2^	40 (28–51)	35 (29–43)	0.35
SV inflow, ml	24 ± 12	26 ± 10	0.39
rel. SV inflow, %	44 ± 8	48 ± 9	**0.02**
RVEF apex, %	42 ± 13	44 ± 14	0.70
EDV apex, ml	49 (29–65)	40 (31–52)	0.20
EDVi apex, ml/m^2^	27 (17–32)	21 (16–26)	0.11
SV apex, ml	21 ± 10	18 ± 8	0.15
rel. SV apex, %	38 ± 10	33 ± 8	**0.01**
RVEF RVOT, %	29 ± 11	32 ± 12	0.27
EDV RVOT, ml	33 (26–42)	32 (26–39)	0.47
EDVi RVOT, ml/m^2^	17 (15–21)	16 (14–19)	0.46
SV RVOT, ml	10 ± 4	10 ± 5	0.55
rel. SV RVOT, %	18 ± 5	19 ± 6	0.76
Apex/inflow rel. SV ratio	0.97 ± 0.51	0.74 ± 0.29	**0.04**

*Values are means ± standard deviations, medians (interquartile ranges) or n (%). p-values are derived from unpaired t-tests, Mann–Whitney U tests or chi-square tests where appropriate. Significant p-values are highlighted in bold*.

*3D-STE, three-dimensional speckle-tracking echocardiography; ECLS, extracorporeal life support; EDV(i), end-diastolic volume (index); FAC, fractional area change derived from 3D-STE; eGFR, estimated glomerular filtration rate; LV-GLS, left ventricular global longitudinal strain; LVAD, left ventricular assist device; LVEDV(i), left ventricular end-diastolic volume (index); LVESV(i), left ventricular end-systolic volume (index); LVEF, left ventricular ejection fraction; OPCAB, off-pump coronary artery bypass surgery; rel., relative; RVEDV(i), right ventricular end-diastolic volume (index); RVESV(i), right ventricular end-systolic volume (index); RVEF, right ventricular ejection fraction; RVOT, right ventricular outflow tract; SV, stroke volume; TAPSE, tricuspid annular plane systolic excursion derived from 3D-STE*.

### Association With Outcome

**Figure 3:**
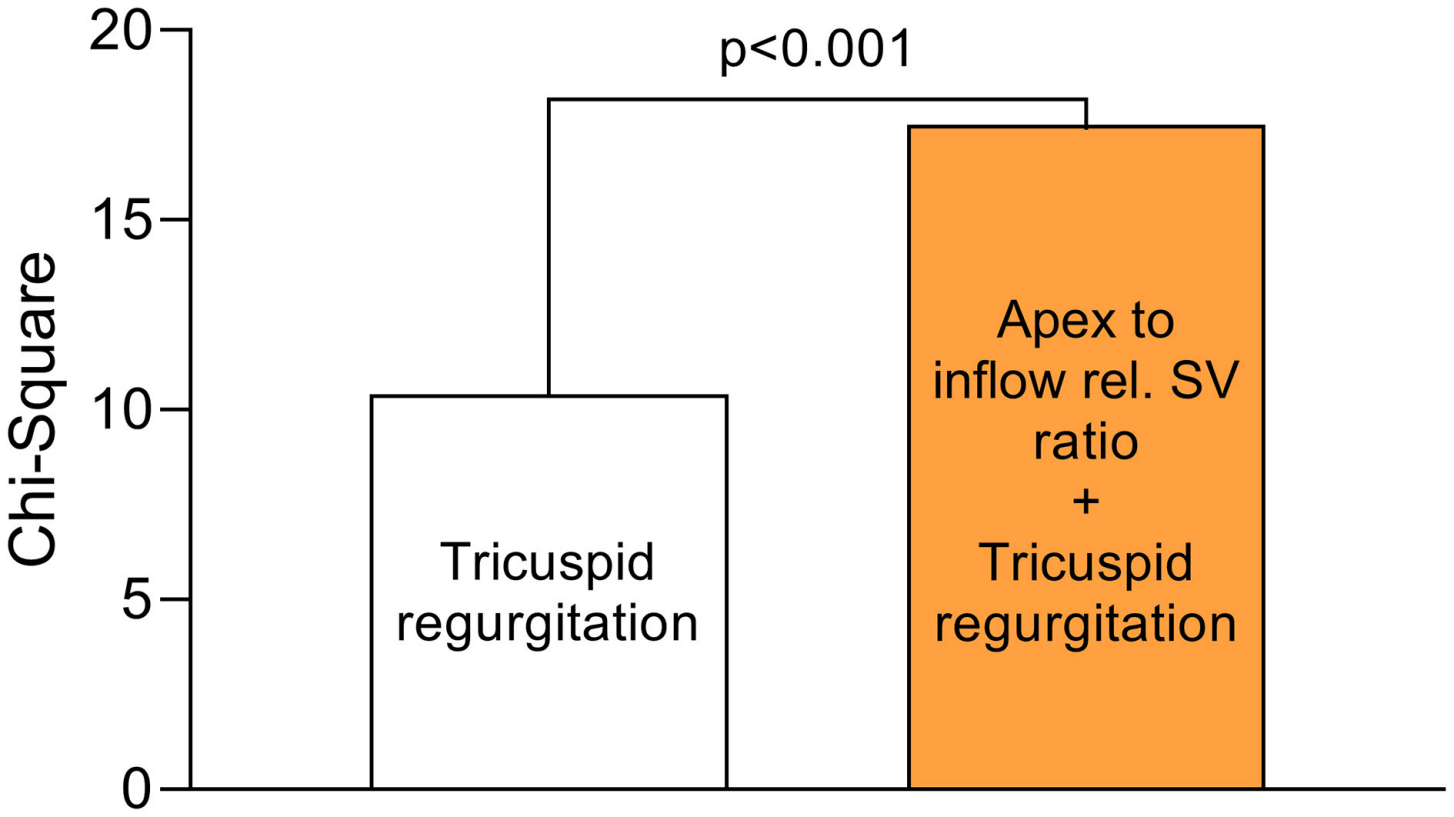
Incremental value of apex to inflow rel. SV ratio over the presence of tricuspid regurgitation. In a multivariable logistic regression model (*n* = 357) for the combined endpoint (in-hospital mortality/ECLS), apex to inflow rel. SV ratio added incrementally to the model over the presence of tricuspid regurgitation (grade ≥ 2). The chi-square values were compared using the omnibus test of model coefficients.

A total of *n* = 25 patients (7%) reached the endpoint. Nineteen patients died during the primary hospital stay (5%), and 11 of those required postoperative ECLS (3%). Six patients required postoperative ECLS but were successfully discharged (2%). The endpoint incidence was 6% (3/51) among on-pump coronary artery bypass grafting patients, 7% (5/69) among OPCAB patients, 8% (8/96) among left-sided valve surgery patients, 6% (1/17) among thoracic aortic surgery patients, 4% (1/25) among LVAD patients and 7% (7/99) among mixed procedure patients. In univariable logistic regression of the investigated clinical and conventional echocardiographic parameters, only tricuspid regurgitation grade ≥ 2 was significantly associated with the endpoint (OR 3.75, 95% CI 1.52–9.30, *p* < 0.01). The odds ratio of a binary variable defines the relative risk change of reaching the endpoint in case the variable is present: e.g., 375% increased risk of patients with tricuspid regurgitation grade ≥ 2 to reach the endpoint compared with patients with tricuspid regurgitation grade 0 or 1. The surgical categories were not significantly associated with the endpoint in univariable logistic regression (Supplementary Table 3). Strikingly, inflow RVEF (OR 0.95, 95% CI 0.92–0.99, *p* = 0.01), rel. SV of the inflow (OR 0.94, 95% CI 0.90–0.99, *p* = 0.02), rel. SV of the apex (OR 1.07, 95% CI 1.02–1.13, *p* < 0.01) and apex to inflow rel. SV ratio (OR 5.81, 95% CI 1.90–17.77, *p* < 0.01) showed a significant association with short-term outcome (Table 3). The OR of a continuous variable defines the relative risk change of reaching the endpoint if the variable increases by one unit: e.g., an increase of inflow RVEF by 1% decreases the risk by 5%, while an increase of apex rel. SV by 1% increases the risk by 7%. In a multivariable regression model including tricuspid regurgitation, inflow RVEF and apex to inflow rel. SV ratio, only tricuspid regurgitation (OR 4.24, 95% CI 1.66–10.84, *p* < 0.01) and apex to inflow rel. SV ratio (OR 6.55, 95% CI 2.09–20.60, *p* < 0.001) remained independently associated with the endpoint. In this model, apex to inflow rel. SV ratio carried incremental value and added significantly to the model fit (Figure 3).

**Table 3:** Univariable and multivariable logistic regression analysis for the association with the endpoint (in-hospital mortality or the need for ECLS).

**Parameter**	**Univariable OR (95% CI)**	***p*-value**	**Multivariable OR (95% CI)**	***p*-value**
Age, years	1.00 (0.97–1.04)	0.91		
Body mass index, kg/m^2^	1.02 (0.95–1.10)	0.62		
Chronic lung disease^*^	2.21 (0.29–16.96)	0.45		
Pulmonary hypertension^†^	0.00 (–)	0.99		
Tricuspid regurgitation^‡^	3.75 (1.52–9.30)	**<0.01**	4.24 (1.66–10.84)	**<0.01**
Diabetes^§^	0.56 (0.18–1.73)	0.31		
NYHA class IV	0.71 (0.27–1.86)	0.49		
eGFR, ml/min	0.99 (0.98–1.01)	0.30		
Lactate, mmol/l	1.84 (0.96–3.52)	0.07		
Hematocrit, %	0.96 (0.90–1.03)	0.23		
EuroSCORE II, %	1.01 (0.96–1.06)	0.79		
LVEDVi, ml/m^2^	1.00 (0.99–1.01)	0.45		
LVEF, %	1.00 (0.97–1.03)	0.94		
LV-GLS, %	1.03 (0.96–1.11)	0.38		
RVESVi, ml/m^2^	1.01 (0.99–1.03)	0.16		
RVEF, %	0.97 (0.93–1.01)	0.10		
FAC, %	0.97 (0.93–1.00)	0.08		
RVEF inflow, %	0.95 (0.92–0.99)	**0.01**	Excluded	0.32
rel. SV inflow, %	0.94 (0.90–0.99)	**0.02**		
RVEF apex, %	0.99 (0.97–1.02)	0.70		
rel. SV apex, %	1.07 (1.02–1.13)	**<0.01**		
RVEF RVOT, %	0.98 (0.95–1.02)	0.27		
rel. SV RVOT, %	0.99 (0.93–1.06)	0.76		
Apex/inflow rel. SV ratio	5.81 (1.90–17.77)	**<0.01**	6.55 (2.09–20.60)	**<0.001**

*CI, confidence interval; eGFR, estimated glomerular filtration rate; FAC, fractional area change; LV-GLS, left ventricular global longitudinal strain; LVEDV(i), left ventricular end-diastolic volume (index); LVEF, left ventricular ejection fraction; NYHA, New York Heart Association; OR, odds ratio; rel., relative; RVESV(i), right ventricular end-systolic volume (index); RVEF, right ventricular ejection fraction; RVOT, right ventricular outflow tract; SV, stroke volume*.

* *Long term use of bronchodilators or steroids for lung disease*.

† *Systolic pulmonary artery pressure > 30 mmHg*.

‡ *Grade ≥ 2*.

§ *Insulin-dependent*.

*Significant p-values are highlighted in bold*.

### Reproducibility

**Figure 4:**
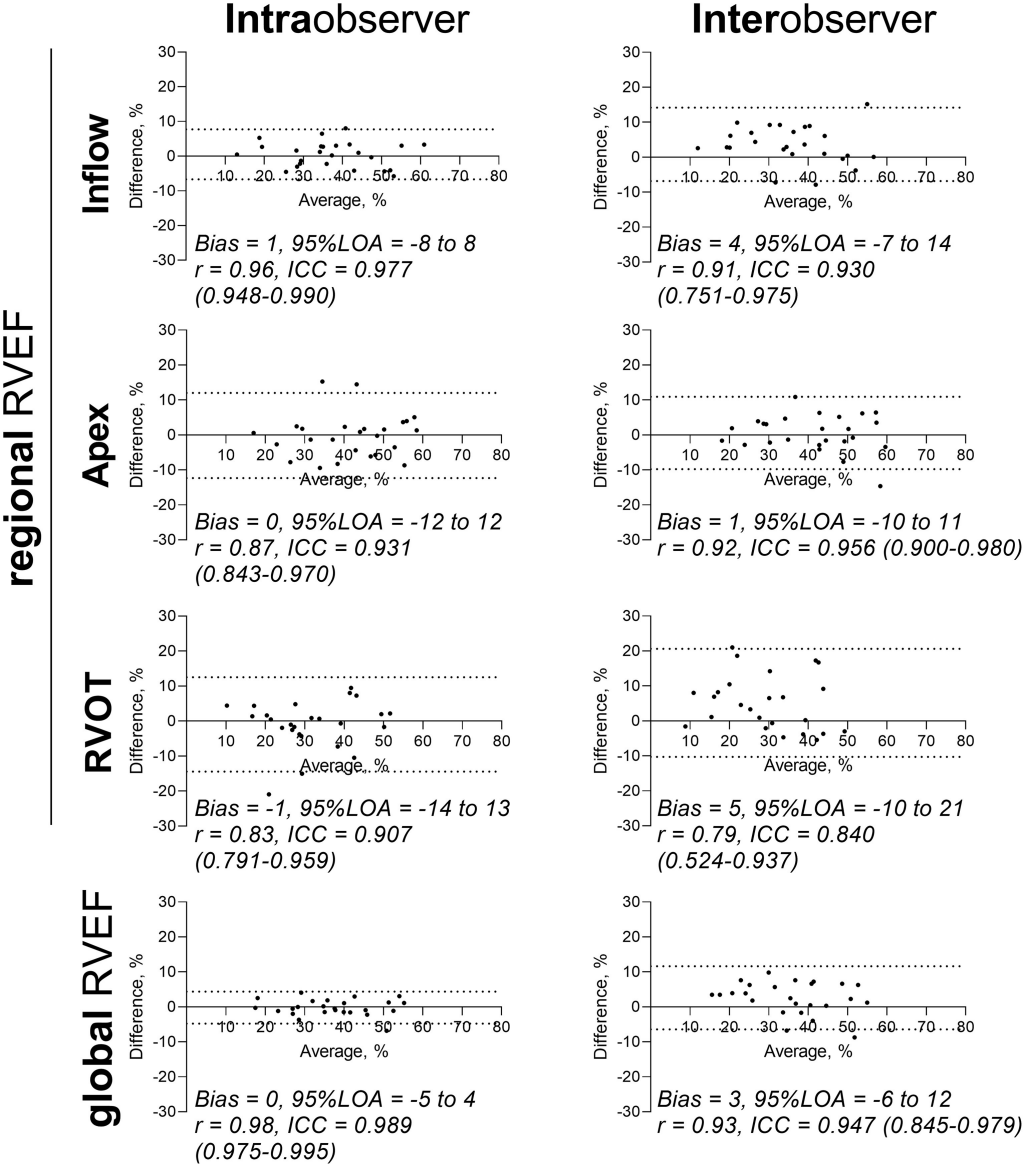
Reproducibility analysis of global and regional RVEF measurements. Intraobserver and interobserver analysis of *n* = 25 patient meshes. Bland–Altman diagrams are displayed for global and regional RVEFs with biases, 95% limits of agreement (95% LOA), correlation coefficients r and intraclass correlation coefficients (ICCs) with 95% confidence intervals. ICC, intraclass correlation coefficient; LOA, limits of agreement; r, Pearson correlation coefficient; RVEF, right ventricular ejection fraction; RVOT, right ventricular outflow tract.

Figure 4 and Table 4 summarize the results of the reproducibility analysis of the double measured patients (*n* = 25). Reproducibility was better for global RVEF analysis than for regional RVEF analysis. While intraobserver and interobserver variability was fairly low for inflow and apex RVEF measurements, RVOT RVEF showed the poorest comparability, with wide 95% limits of agreement (−10 to 21) and an ICC of 0.840 (95% CI: 0.524–0.937). Interobserver variability of apex to inflow rel. SV ratio was moderate [*r* = 0.75, *p* < 0.0001; ICC 0.820 (0.591–0.920); bias −0.02; 95% limits of agreement −0.34 to −0.30].

**Table 4:** Intra- and interobserver reproducibility analysis for global and regional RVEF.

**Parameter**	***r***	**ICC (95% CI)**	**Bias (%)**	**95% LOA (%)**
**Intraobserver reproducibility**				
RVEF inflow, %	0.96	0.977 (0.948–0.990)	1	−8 to 8
RVEF apex, %	0.87	0.931 (0.843–0.970)	0	−12 to 12
RVEF RVOT, %	0.83	0.907 (0.791–0.959)	−1	−14 to 13
RVEF, %	0.98	0.989 (0.975–0.995)	0	−5 to 4
**Interobserver reproducibility**				
RVEF inflow, %	0.91	0.930 (0.751–0.975)	4	−7 to 14
RVEF apex, %	0.92	0.956 (0.900–0.980)	1	−10 to 11
RVEF RVOT, %	0.79	0.840 (0.524–0.937)	5	−10 to 21
RVEF, %	0.93	0.947 (0.845–0.979)	3	−6 to 12

*CI, confidence interval; ICC, intraclass correlation coefficient; LOA, limits of agreement; r, Pearson correlation coefficient; RVEF, right ventricular ejection fraction; RVOT, right ventricular outflow tract*.

## Discussion

### Key Findings

In a mixed cohort of elective cardiac surgery patients, most baseline clinical and intraoperative echocardiographic parameters showed no significant association with short-term outcome, including sophisticated measures such as 3D-derived RVEF and LV-GLS. Strikingly, an established score for perioperative risk assessment (EuroSCORE II) did not correlate with adverse outcomes in these patients. Using a simple approach to quantify regional systolic function in three anatomically different regions of the RV, a novel parameter incorporating the relative contributions of the apex and the inflow tract to volume ejection (apex to inflow rel. SV ratio) was significantly and independently associated with the combined endpoint of in-hospital death and life-threatening hemodynamic failure requiring ECLS. Only the presence of significant tricuspid regurgitation (grade ≥ 2) showed similar associations in logistic regression. Furthermore, the novel technique for regional RV analysis is feasible and reproducible, with higher observer variabilities in the RVOT region, potentially because of its complex anatomy and relatively small volume. Reproducibility was further warranted by an identical definition of the segmental borders in all meshes, avoiding errors due to manual border determination. Overall, parameters of cardiac function acquired intraoperatively using novel 3D techniques were lower than conventional reference ranges, a phenomenon that has been discussed previously (20).

### Perioperative Right Ventricular Function

Assessment of systolic RV function by intraoperative TEE is crucial. The induction of general anesthesia and positive pressure ventilation have distinct effects on RV function (21), and the application of goal-directed therapies by the anesthesiologist—such as vasopressor infusions to achieve predefined mean arterial pressures—could help to equilibrate loading conditions. Baseline reductions in RV function are associated with perioperative RV dysfunction and adverse outcomes following cardiac surgery (22), but the sensitivities of traditional echocardiographic measures are potentially insufficient (23). TAPSE, e.g., quantifies longitudinal function of the basal inflow segment but did not correlate with the endpoint, in contrast to the novel inflow parameters. Preoperative TTE-derived 2D TAPSE measurements might yield different results. The paradigm of disease-induced RV enlargement and dilation with increased EDVs has existed for decades. However, RVEDV showed no independent association with short-term outcomes in a previous investigation from our institution (20). Another possible phenotype of pathological RV remodeling could be reflected by altered regional EDVs in the absence of global enlargement, e.g., increased apical EDVs vs. decreased inlet EDVs. In conditions of pressure or volume overload, regionally different adaptive reactions according to the heterogeneous structure within the right ventricular myocardium appear plausible (24). However, in our retrospective analysis, regional EDVs did not differ significantly between patients with good and unfavorable outcomes. Instead, our data stress the hypothesis that a deterioration of RV function precedes enlargement of the RV, as regional functional parameters (e.g., rel. SV) of the regions showed distinct differences between the outcome groups. Rel. SV is further affected by the volume of a segment and should be considered as a parameter associated with RV remodeling. Consequently, the assessment of static parameters or the quantification of altered shapes might be less favorable than novel functional measures in the context of elective cardiac surgery. During the analysis of our findings, we intuitively linked them to a historic echocardiographic finding named McConnell's sign: during acute pressure overload or ischemia, the stressed RV in some cases reacts with a distinct contraction pattern (25, 26). While the apex shows normal or even hyperdynamic contractility, the basomidventricular free wall myocardium appears hypo- or akinetic (27). Even though McConnell's sign is usually observed during acute RV failure, the shift from inflow to apical relative stroke volume ejection in our patients with adverse short-term outcomes could point to a mild or chronic form of a *McConnell-esque* contraction pattern, indicating subclinical RV dysfunction.

### Risk Stratification in Cardiac Surgery and Clinical Implications

The prediction of postoperative outcomes following cardiac surgery is important but challenging. If the onset or progression of cardiovascular disease indicates surgery, interdisciplinary teams involving cardiac surgeons, cardiologists and anesthesiologists need to decide for or against the procedure based on the individual benefit-risk assessment. Currently, a wide variety of clinical features are available, but their interpretation and integration are complex. Established current risk scores, such as the EuroSCORE II or the Society of Thoracic Surgeons Risk Score, have been shown to predict unfavorable postoperative outcomes but do not incorporate a direct measure of RV systolic function (28, 29). Therefore, these scoring systems are prone to severely underestimating patients' risks in cases of RV dysfunction. In the context of cardiac anesthesia, the interpretation of intraoperative TEE-derived functional parameters—among other parameters of respiratory function or invasive hemodynamics, for example—is paramount for evaluating the patients' needs regarding postoperative care (30). While the course of an operation and its surgical success can be similar, patient outcomes can vary dramatically depending on their ability to recover from major surgical trauma, extracorporeal circulation or altered loading conditions. Hence, modern perioperative strategies focus on the individual patient's needs and range from fast-track regimens to extensive ICU protocols. While short durations of mechanical ventilation and early mobilization decrease the risk for postoperative complications in eligible patients (31), the identification of patients at risk for hemodynamic failure is important and should lead to a continuous evaluation of diagnostic measures and subsequent therapies—such as inotropic support or ECLS—if necessary (32). Our results indicate that sophisticated measures of RV function, measured via intraoperative TEE by the anesthesiologist, have the potential to predict adverse short-term outcomes. As these parameters, e.g., apex to inflow rel. SV ratio, are not part of current and established risk scores, their assessment should be considered during the planning and initiation of the postoperative regimen. Future studies should attempt to unveil the potential of this parameter in preoperative risk assessment prior to cardiac surgery employing awake transthoracic echocardiography. Technical optimizations of regional RV volumetry to warrant online, bedside applicability are needed to facilitate clinical implementation.

### Limitations

Naturally, there are some limitations to the interpretation of the results and their generalizability. The investigated patients were retrospectively included, resulting in a strictly observational study design. As not all patients underwent intraoperative 3D echocardiography, a potential inclusion bias needs to be considered during interpretation of the results. Furthermore, incidence of the endpoint is low resulting in a limited number of events. Prospective randomized trials are necessary to fully characterize the association between the reported parameters and patient outcomes following cardiac surgery. Baseline cardiac function was not characterized by other techniques, such as invasive hemodynamics, awake transthoracic echocardiography or cardiac magnetic resonance imaging. Hence, a comparison of the novel measures with reference methods cannot be provided. Awake pulmonary artery pressures and the underlying cause of present pulmonary hypertension might be associated with the outcome but were not systematically available in this cohort. The severity of tricuspid regurgitation was a variable of interest in regression analysis but the reproducibility of the assessment of tricuspid regurgitation was not investigated systematically in this cohort. Since only the association of clinical and echocardiographic parameters with short-term outcome was studied, the report does not allow for conclusions regarding long-term prognosis.

### Conclusions

Regional right ventricular systolic function assessed by intraoperative three-dimensional transesophageal speckle-tracking echocardiography is feasible. The apex to inflow relative stroke volume ratio is associated with increased in-hospital mortality and life-threatening postoperative hemodynamic failure. These findings might be useful to improve perioperative risk estimation in patients with baseline RV dysfunction in which conventional echocardiographic parameters are within normal range.

## Data Availability Statement

The raw data supporting the conclusions of this article will be made available by the authors, without undue reservation.

## Ethics Statement

The studies involving human participants were reviewed and approved by Ethics Committee of the Medical School at the University of Tuebingen, Gartenstraße 47 72074 Tuebingen, Germany. Written informed consent for participation was not required for this study in accordance with the national legislation and the institutional requirements.

## Author Contributions

MK and HM contributed to conception and design of the study. MK, M-MD, and TH performed data acquisition. MK and HM performed the statistical analysis. MK, AK, CS, PR, and HM performed data interpretation. MK wrote the first draft of the manuscript. AK, CS, and PR critically revised the manuscript. HM contributed substantially to the final revision of the manuscript. All authors read and approved the submitted version.

## Funding

This work was supported by Deutsche Forschungsgemeinschaft (DFG) (Grant DFG-INST 2388/71–1 FUGG) and funded by departmental funds.

## Conflict of Interest

The authors declare that the research was conducted in the absence of any commercial or financial relationships that could be construed as a potential conflict of interest.

## Publisher's Note

All claims expressed in this article are solely those of the authors and do not necessarily represent those of their affiliated organizations, or those of the publisher, the editors and the reviewers. Any product that may be evaluated in this article, or claim that may be made by its manufacturer, is not guaranteed or endorsed by the publisher.

## Abbreviations

3D: three-dimensional
CI: confidence interval
ECLS: extracorporeal life support
EDV(i): end-diastolic volume (index)
ESV(i): end-systolic volume (index)
eGFR: estimated glomerular filtration rate
FAC: fractional area change
ICC: intraclass correlation coefficient
LVAD: left ventricular assist device
LVEF: left ventricular ejection fraction
LV-GLS: left ventricular global longitudinal strain
NYHA: New York Heart Association
OPCAB: off-pump coronary artery bypass surgery
OR: odds ratio
rel.: relative
RV: right ventricle/right ventricular
RVEDV(i): right ventricular end-diastolic volume (index)
RVEF: right ventricular ejection fraction
RVESV(i): right ventricular end-systolic volume index
RVOT: right ventricular outflow tract
STE: speckle-tracking echocardiography
SV: stroke volume
TAPSE: tricuspid annular plane systolic excursion
TEE: transesophageal echocardiography/echocardiograms.
