# Supplementary material for: Regional Right Ventricular Function Assessed by Intraoperative Three-Dimensional Echocardiography Is Associated With Short-Term Outcomes of Patients Undergoing Cardiac Surgery
Source: Front Cardiovasc Med. 2022 Mar 22;9:821831. doi: 10.3389/fcvm.2022.821831 (PMC8980927; doi:10.3389/fcvm.2022.821831)
Supplement: Supplementary file 1 [file Data_Sheet_1.docx]

Suppl. Table 1: Procedures categorized as “mixed”

| **Procedures** | **n (%)** |
| --- | --- |
| CABG + AVS | 23 |
| AVS + Thoracic aortic surgery | 21 |
| CABG + MVS | 16 |
| MVS + TVS | 8 |
| MVS + LAA Occlusion | 7 |
| CABG + Thoracic aortic surgery | 5 |
| AVS + MVS + Thoracic aortic surgery | 3 |
| CABG + AVS + MVS | 2 |
| CABG + AVS + Thoracic aortic surgery | 2 |
| CABG + Dor procedure | 2 |
| MVS + Thoracic aortic surgery | 2 |
| AVS + Myectomy | 1 |
| CABG + TVS | 1 |
| MVS + ASD closure | 1 |
| MVS + PFO closure | 1 |
| MVS + TVS + Thoracic aortic surgery | 1 |
| Thoracic aortic surgery + ASD closure | 1 |
| Thoracic aortic surgery + Myectomy | 1 |
| Thoracic aortic surgery + RVOT reconstruction | 1 |

ASD = atrial septal defect, AVS = aortic valve surgery, CABG = coronary artery bypass grafting, LAA = left atrial appendage, MVS = mitral valve surgery, PFO = patent foramen ovale, RVOT = right ventricular outflow tract, TVS = tricuspid valve surgery

Suppl. Table 2: Global and regional RVEF in patient groups categorized according to systolic pulmonary artery pressures (sPAP)

| **sPAP:** | **<31mmHg** | **31-55mmHg** | **>55mmHg** | **p value** |
| --- | --- | --- | --- | --- |
| **RVEF, %** | 38 ± 10 | 34 ± 11 | 30 ± 11 | <0.001 |
| **RVEF RVOT, %** | 33 ± 11 | 27 ± 12 | 24 ± 14 | <0.001 |
| **RVEF inflow, %** | 38 ± 11 | 36 ± 12 | 30 ± 13 | <0.01 |
| **RVEF apex, %** | 45 ± 13 | 38 ± 15 | 37 ± 17 | <0.01 |

Values are means ± standard deviations. P values were computed with one-way ANOVA.

RVEF = right ventricular ejection fraction, RVOT = right ventricular outflow tract, sPAP = systolic pulmonary artery pressure

**Suppl. Table 3: Univariable logistic regression analysis for the association of the procedure types with the endpoint (in-hospital mortality or the need for ECLS)**

| **Parameter** | **OR (95% CI)** | **p- value** |
| --- | --- | --- |
| **On-pump coronary artery bypass grafting** | 0.81 (0.23-2.80) | 0.74 |
| **OPCAB** | 1.05 (0.38-2.90) | 0.93 |
| **Left-sided valve surgery** | 1.30 (0.54-3.13) | 0.55 |
| **Thoracic aortic surgery** | 0.82 (0.10-6.47) | 0.85 |
| **LVAD implantation** | 0.53 (0.07-4.12) | 0.55 |
| **Mixed procedures** | 1.01 (0.41-2.51) | 0.98 |

CI = confidence interval, ECLS = extracorporeal life support, LVAD = left ventricular assist device, OPCAB = off-pump coronary artery bypass surgery, OR = odds ratio, rel. = relative
